# Predator-prey interactions in the plankton: larval fish feeding on evasive copepods

**DOI:** 10.1038/srep33585

**Published:** 2016-09-23

**Authors:** James M. Jackson, Petra H. Lenz

**Affiliations:** 1 Békésy Laboratory of Neurobiology, Pacific Biosciences Research Center, University of Hawaii at Manoa, 1993 East-West Rd., Honolulu, HI 96822, USA.

## Abstract

Capture success and prey selectivity were investigated in clownfish *Amphiprion ocellaris* larvae using videography. Three prey types were tested using developmental stages (nauplii, copepodites and adults) of the copepod *Parvocalanus crassirostris*. Predatory abilities improved rapidly between days 1 and 14 post-hatch. Initially, capture success was limited to nauplii with few attacks on larger stages. Captures of copepodites were first observed at 3 dph, and of adults at 8 dph. Consistent strikes at the larger prey were observed on the day prior to successful captures (2 dph for copepodites, 7 dph for adults). Difference in capture success between nauplii and adults at 8 dph was an order of magnitude. Differences in capture success among prey types persisted but decreased to three-fold by 14 dph. Younger *A. ocellaris* attacked nauplii preferentially and avoided adult prey. Strike selectivity declined with age, and no selectivity was observed after 10 dph. However, numerically 50% of the ingested prey were still nauplii at 14 dph under the experimental conditions.

The planktonic larval phase of fishes is characterized by high recruitment variability and mortality rates that exceed 99%^1^. While multiple factors contribute to this variability, the inability to find and capture sufficient food during the planktonic phase is one factor that can lead to poor growth and increased mortality^2^,^3^,^4^,^5^,^6^. Marine larval fishes are typically zooplanktivorous, feeding on a variety of micro- and meso-zooplankton as shown by gut content analysis^7^,^8^,^9^,^10^,^11^. Although food availability may not be limiting in some habitats^8^,^12^, faster growth rates are correlated with higher recruitment success in both temperate and coral reef larval fishes^2^,^13^,^14^. Furthermore, growth rates are often correlated with prey abundances^6^,^15^. Larvae of sub-tropical reef fishes have higher energetic demands^13^ and swimming endurances in late stage planktonic fish larvae appear to be related to feeding status, suggesting that availability of prey may be critical for larvae near settling^16^.

The diets of wild-caught larval fishes are often dominated by copepods as indicated by gut content analyses^9^,^12^,^17^,^18^,^19^. However, feeding success on evasive prey can be low in larval fishes^20^,^21^, which could effectively lower the number of available prey, at least in early stage larval fishes^22^,^23^. Here, we investigated the changes in capture success and prey selectivity between first feeding and age of settlement in a predator-prey system that serves as a model for coral reef systems: the larvae of the clownfish, *Amphiprion ocellaris* preying on different developmental stages of the sub-tropical calanoid copepod *Parvocalanus crassirostris*.

## Copepod Prey

Copepods are small crustaceans that inhabit marine and freshwater environments. Free-living planktonic forms range in size from 0.1 to 10 mm in length, and they are an important source of food for invertebrates, fishes, birds and even marine mammals^24^,^25^,^26^. Copepods are also among the most evasive planktonic organisms in the pelagic environment^27^. They possess an array of mechanosensory setae on the first antenna, which are sensitive to hydromechanical disturbances^28^,^29^,^30^,^31^,^32^,^33^. Escape responses are characterized by high accelerations and swimming speeds (>100 m s^−2^, and >350 m s^−1^ for a 1 mm copepod) and very short response latencies (<3 ms)^34^,^35^,^36^,^37^,^38^,^39^.

The copepod prey in this study was a small sub-tropical species, the paracalanid, *Parvocalanus crassirostris*, which ranged in size from 60 to 400 μm in length^40^. Behavioral sensitivity and escape behaviors of multiple developmental stages (nauplii and copepodites) of *P. crassirostris* have been quantified using an artificial stimulus^41^. The early developmental stages, nauplii, were found to be less sensitive to the hydromechanical stimulus, and their behavioral latency was longer than those of the copepodites (4 vs. 3 ms)^41^. Maximum escape speeds increased with copepod size from 36 to >150 mm per second and scaled with length^41^. All developmental stages of this copepod had well-developed escape responses, and maximum swim speeds are similar to those reported for 3 to 8 mm larval fish (~60 to 200 mm s^−1^)^42^,^43^.

### Larval fish predatory behavior

Predatory behavior of fish larvae involves a slow approach while bending of the tail followed by a sudden forward thrust and capture of prey^22^,^44^. Feeding success increases with development: larvae increase their swimming speeds, and their approach is less likely to elicit an escape response in the prey^21^,^44^. Although copepods are typically found in larval fish guts, fish larvae such as cod (*Gadus morhua*) may not start feeding on even the youngest copepod stages until nine days post-hatch, four to five days after first feeding, preferring to feed initially on protozoa, which swim more slowly and have limited escape responses^21^. Thus, while low encounter rates with prey may contribute to low feeding rates^6^,^45^,^46^, capture success may be equally important, in particular at first feeding when predatory abilities are still poorly-developed, even for non-evasive prey^5^,^21^,^44^,^47^.

### Clownfish-copepod interaction

The planktonic larval phase of clownfish (*Amphiprion*: Pomacentridae) spans approximately two weeks from hatching, which is representative of the duration of the larval phase of many reef fishes^48^. In contrast to many temperate fish larvae, clownfish feed on copepods and phytoplankton through most of the larval period^49^, although not much is known about their feeding success. As newly hatched larvae, clownfish carry a yolk sac, which is fully absorbed by the third day post-hatch^50^. During the first two days, feeding rates in at least some species is very low, and capture success on non-evasive rotifers is less than 60%^47^,^51^. Captive rearing protocols have been developed for several clownfish species, including *A. ocellaris*, used in this study^52^.

Zooplankton communities in many sub-tropical environments are dominated by small copepod species (≤1 mm in length) in the families Paracalanidae (Calanoida), Clausocalanidae (Calanoida), and Oithonidae (Cyclopoida)^53^,^54^,^55^,^56^,^57^,^58^. The paracalanid *P. crassirostris* has a widespread distribution and occurs in coastal waters in the Atlantic, Indian and Pacific Oceans^53^,^54^,^55^,^56^,^57^,^58^,^59^,^60^. In much of its geographic range, this species co-occurs with members of the Pomacentridae, including *A. ocellaris* and other clownfish species^61^,^62^.

*P. crassirostris* is similar in size, body form, life history and ecology to other small paracalanids that dominate many sub-tropical habitats^61^,^63^. Gut content analysis studies of coral reef fish larvae usually classify calanoid copepod prey by developmental stage (nauplii, copepodites) or size (small vs. large species)^9^,^18^, where paracalanid prey, such as *P. crassirostris* would be among the “nauplii”, “copepodites” and “small calanoids” categories. According to these studies larval Pomacentridae consume copepod nauplii, copepodites and small calanoids but not large copepods^9^,^18^.

Here, we investigated growth, capture success and prey selectivity of larvae of the clownfish, *Amphiprion ocellaris* under experimental conditions using video recordings of predator-prey interactions between larval fish and copepod prey that ranged in size and evasive capabilities. Nauplii, copepodites and adults, i.e., different developmental stages of the copepod *P. crassirostris* were used as prey. While prey selectivity is usually determined from gut contents, the focus of this study was to quantify which prey types were preferentially attacked and determine the outcome of the interaction. While predator’s mouth gape has been used to predict prey size^64^,^65^,^66^,^67^, this may not apply when highly evasive zooplankton prey are considered. Specifically, we hypothesized that changes in capture success and prey selectivity between first feeding and age of settlement could not be explained by mouth gape. Thus, the goal was to quantify predatory abilities of a larval fish during its planktonic phase in order to understand how feeding success on a natural prey changed during early development.

## Results

### Amphiprion ocellaris growth

**Figure 1:**
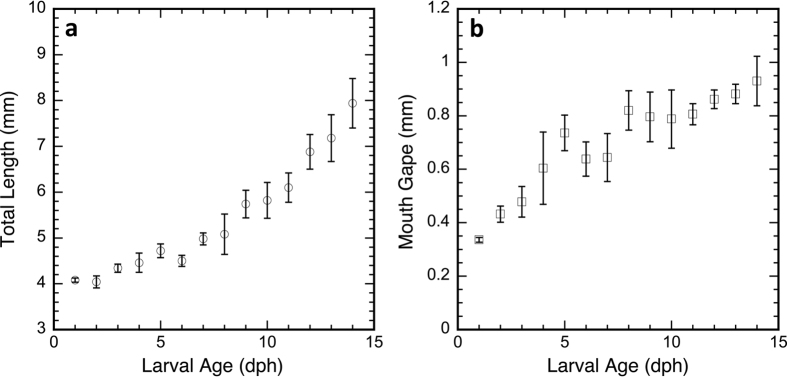
Growth of *Amphiprion ocellaris* larvae during the first two weeks post-hatch. (**a**) Average total length for six individuals for each time point between 1 and 14 dph. (**b**) Average mouth gape. Error bars: standard deviations.

During the first two weeks post-hatch, the larvae nearly doubled their total length from 4.1 mm at 1 day post-hatch (dph) to 7.9 mm at 14 dph (Fig. 1a), while mouth gape increased by a factor of 3 from 0.3 to 1 mm during this time period (Fig. 1b). By 2 dph, the mouth gape exceeded the prosome length of the largest prey (~400 μm, adult female).

### Capture success as a function of larval age

Capture success was investigated in two experiments using three prey types that differed in evasiveness: *P. crassirostris* nauplii, copepodites and adults. In Experiment I, feeding trials consisted of single prey types, and *A. ocellaris* predatory success was determined on days 1, 3 and 10 dph. In Experiment II, feeding trials consisted of mixed prey assemblages and predatory success and prey selectivity were determined daily between 1 and 14 dph. Under our experimental conditions, rejection of prey was not observed and all successful captures led to ingestion of prey (100% ingestion rate).

**Figure 2:**
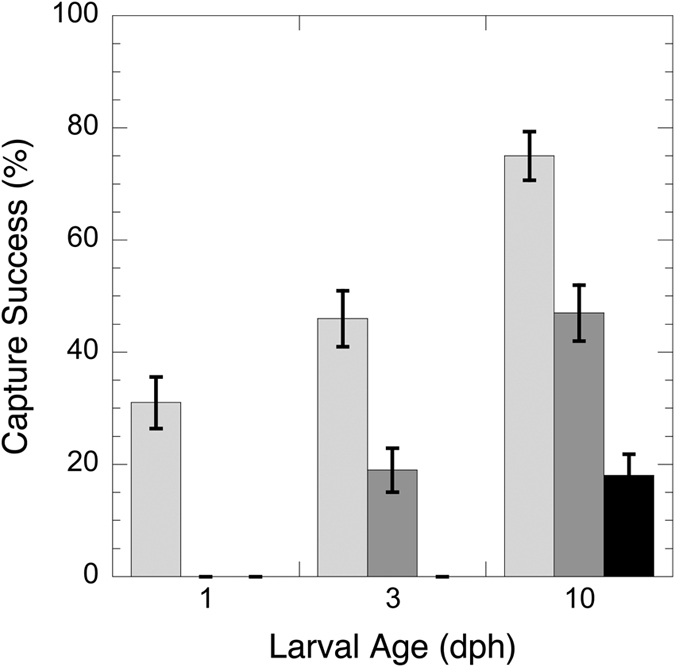
Capture success rate of nauplii (light gray), copepodites (dark gray) and adult (black) *Parvocalanus crassirostris* by *Amphiprion ocellaris* at 1, 3 and 10 dph in Experiment I. Number of observations = 100, except for attacks on adults at 1 dph which resulted in fewer than 25 observed strikes. Single prey type added at an initial density of 1 ml^−1^. Error bars: standard deviations.

Prey type and larval age both had a significant effect on strike outcome in both experiments (p < 0.005; Tables 1 and 2). For Experiment I, *A. ocellaris* prey capture success in behavioral trials with single prey at 1, 3, and 10 dph was always higher on nauplii than for the other prey types, and capture success rates increased with larval age. Initially, larvae were capturing nauplii with a 31% success rate, but unable to capture either copepodites or adults. At 3 dph, the larvae were capturing both nauplii and copepodites at 46% and 19% success rate, respectively, but not adults. At 10 dph, all three prey types were captured, although success rates differed by a factor of four between adult and naupliar prey types (Fig. 2).

**Figure 3:**
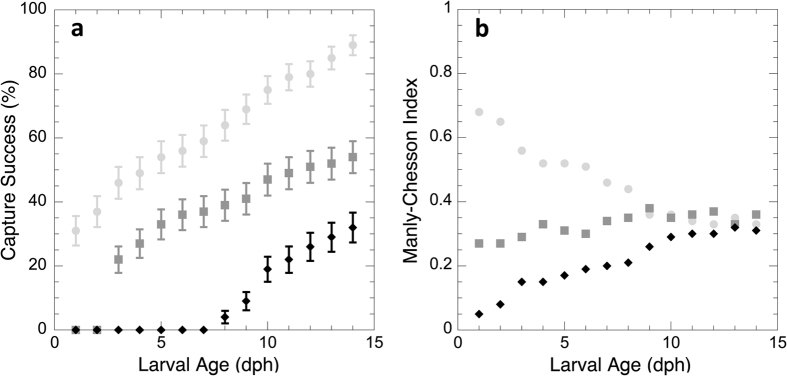
Capture success rate and prey selectivity of *Amphiprion ocellaris* between 1 and 14 dph on nauplii, copepodites and adults of *Parvocalanus crassirostris* in Experiment II. (**a**) Capture success rate on nauplii (light gray circles), copepodites (dark gray squares), and adults (black diamonds) in mixed prey assemblages with 1 prey ml^−1^ in equal proportions by prey type. Number of observations = 100 strikes per prey type and dph, except for days 1 to 6 dph for adult prey, and 1 dph for copepodite prey. Error bars: standard deviations. (**b**) Manly-Chesson index calculated for each day post-hatch. Nauplii: light gray circles; copepodite: dark gray square; adults: black diamonds.

Similar results were obtained in Experiment II using mixed prey assemblages (Fig. 3a). This experimental series confirmed that fish larvae captured only nauplii initially. Successful capture of copepodites was first observed at 3 dph, and successful capture of adults was first observed at 8 dph (Fig. 3a). Predatory capability increased with age as shown by the successive addition of copepodites and adults to the capture repertoire, as well as a steady increase in capture success for each prey type. Thus, in these experiments, the larval fish captured only nauplii and copepodites during pre-flexion, and added adult prey after reaching post-flexion at 7 dph. By 14 dph, capture success rates were ca. 90% for nauplii, 50% for copepodites and 30% for adults (Fig. 3a). A regression analysis for the period that nauplii, copepodites and adults were captured (7 to 14 dph) showed significant effects (p < 0.005) on strike outcome for the three prey types (Table 2).

### Prey selectivity

Predation selectivity, defined as a pattern of predator preference or avoidance for striking a prey type that cannot be explained by the probability of encountering that prey type in the environment, was quantified via the Manly-Chesson Selectivity Index for each larval age (Fig. 3b). Since the three prey types were at equal densities, a value close to 1/3 indicates no selective behavior in strike rates by the predator. Strikes on copepodite prey were close to this predicted 1/3 for the entire 14-day period, even though these prey were not captured until day 3. However, selective feeding occurred for the other two prey types. Initially, larvae preferentially attacked nauplii while avoiding adults, but the preference for nauplii and avoidance of adults decreased during the larval period, approaching 1/3 after 10 dph. A Pearson chi-square analysis confirmed that strikes on nauplii were significantly different (p < 0.05) from 1/3 between days 1 and 8 post-hatch, but not between days 10 and 14 post-hatch. The observed proportion of strikes on adult copepods was also significantly different (p < 0.05) from 1/3 between days 1 and 9 post-hatch.

## Discussion

The interaction between a predator and a prey can be described as a process that involves multiple steps from search and encounter to strike, capture, and ultimately ingestion^45^,^68^. The high densities of prey (1 prey per ml) in the current study assured high encounter rates between predator and prey, and thus, did not examine search behavior, but focused on capture success. While copepods are the preferred prey of many, if not most marine larval fishes, they are also among the most evasive zooplankters^35^,^36^,^41^,^69^. Thus, early fish larvae may require much higher ambient plankton abundances than would be predicted from ingestion rates and search volumes due to low feeding efficiency^23^. Here, we investigated two aspects of feeding efficiency: prey choice and capture success of a larval fish predator between first preying and settlement preying on a small calanoid copepod with a well-described and quantified escape response.

Predatory success in the clownfish larvae was characterized by a steady increase in capture rates with age for each prey type. The capture success rates reported here (none to 90%) are comparable to those reported in the literature for marine fish larvae feeding on either non-evasive (*Artemia* and rotifers) or evasive (mixed plankton, nauplii) prey. Depending on larval age and fish species reported rates of capture success range from 6% to over 90%^20^,^21^,^22^,^23^,^44^,^70^. However, not all fish larvae ingest copepod nauplii initially, feeding instead on smaller auto- and heterotrophs^21^,^71^,^72^. While improvements in capture efficiency/success of fish larvae have been established for both non-evasive and evasive prey^22^,^23^,^44^,^70^, no studies have quantified how this capture success changes over time using natural prey. *A. ocellaris* added prey during the planktonic phase from a single prey type (nauplii) and modest capture success (~30%) at first feeding to all three prey types by 8 dph. Energetic demands increase steeply during the larval period, and larvae increase the number of prey consumed and ingest larger prey^44^,^70^. In our experiments, which focused on a single, small calanoid species, the addition of larger prey to the diet was the result of changes in strike selectivity and increase in capture success. Nevertheless, in *A. ocellaris* differences in capture success which were as much as 15-fold between nauplius and adult prey at 8 dph suggest that capture of large prey came at a cost. While the difference in capture success between nauplii and adults persisted through development, it declined to 3-fold by 14 dph.

Fish larvae are not only characterized by rapid growth, but also many other developmental changes including the maturation of the sensory, motor, and digestive systems^50^,^73^,^74^. All of these factors are likely contributors to improvements in predatory behavior. In addition, learning may be a factor as well, as suggested by the observation of consistent, yet unsuccessful strikes on copepodites and adults on the day prior to their successful capture. In the current study, the fish larvae were exposed to different developmental stages of *P. crassirostris* in the rearing tank, and this may have contributed to their ability to capture the prey as observed in the behavioral experiments. Other studies have also suggested that experience and learning contribute to feeding success^23^,^44^.

Based on mouth gape measurements, *A. ocellaris* would have been expected to feed on adult *P. crassirostris* by day 2–3 post-hatch. Thus, fish gape, which has been used as a predictor for the maximum size of prey^64^,^65^,^66^,^67^,^75^ was not a valid predictor of prey consumption in this study. In contrast, non-evasive prey, such as rotifers (lorica length: 100–300 μm), which are similar in size to *P. crassirostris* copepodites, are captured with a 100% success rate at 3 dph, as was shown experimentally for the congener, *Amphiprion periderion*^47^. While it has been suggested that the physical development of the larval fish feeding apparatus could limit prey size to less than 50% of gape width^76^, escape capabilities by potential prey may be equally important given the differences in capture success that are independent of prey size.

**Figure 4:**
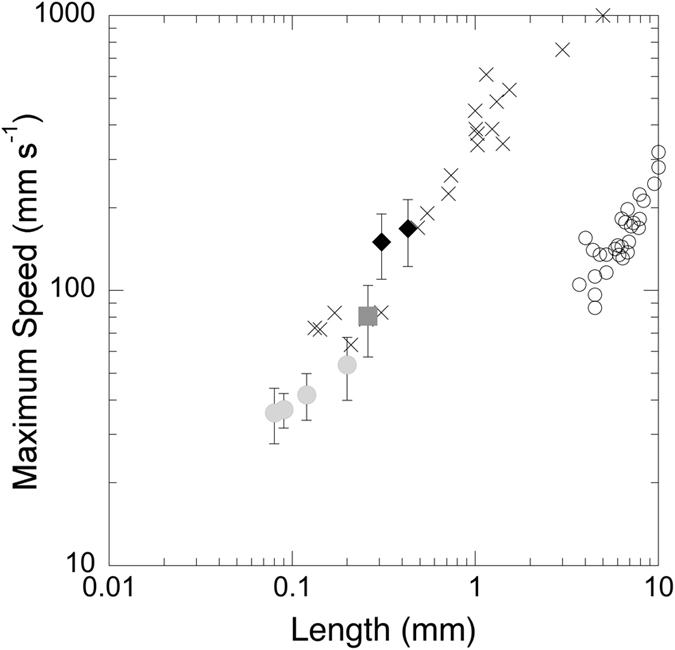
Maximum escape speeds plotted against total length for calanoid copepods, including *Parvocalanus crassirostris* and larval fishes. Maximum swimming speeds for *P. crassirostris* are averages and error bars are standard deviations (nauplii: light gray circles; copepodite: dark gray square; adults: black diamonds)^41^. Crosses: other published copepod data^35^,^36^,^38^,^41^. Open circles: larval fish data are maximum average swim speeds for individuals from five species ranging in length from 3.7 and 10 mm^42^.

Calanoid copepods detect approaching predators using sensitive mechanoreceptors located on the first antenna^29^,^30^,^32^,^77^. The presence of large axons and myelin ensures that response latencies are in the millisecond range^39^,^78^, and that escapes are characterized by rapid acceleration, high speeds and large force production^35^,^36^,^37^,^41^. For all developmental stages of planktonic copepods, maximum escape speeds scaled by length are nearly an order of magnitude higher than those of fishes^38^,^41^,^42^,^79^. In Fig. 4, maximum swim speeds are plotted against length for different species of fish larvae (length: 3–12 mm) and for calanoid copepods, including *P. crassirostris*. Based on the relative sizes of the larval predator (4–8 mm) and the prey, maximum swim speeds of nauplii and early copepodites (CI) would be lower than those predicted for *A. ocellaris* larvae. However, maximum swim speeds of adult *P. crassirostris* are higher than those predicted for the smaller and younger *A. ocellaris* larvae (<6–7 mm; Figs 1 and 4). Thus, initial capture success of adult copepods by the fish larvae (8 dph) occurs before they have reached comparable maximum swim speeds. For a successful strike, the larval fish has to get close enough to the prey without alerting it to the predator’s presence^21^.

Although maximum escape speeds may be an important factor in promoting successful escapes by the prey, sensory detection also plays a significant role in alerting copepods to approaching prey^80^,^81^. Compared with adults, naupliar stages have lower mechanosensitivity, and longer delays to behavioral responses^41^. On average, *P. crassirostris* copepodites responded at a greater distance from the stimulus (2.8 vs. 3.8 mm) with a shorter latency (3 vs. 4 ms) and reached maximum swim speeds (~5 vs. >10 ms) more quickly than nauplii in response to an artificial stimulus^41^. These differences in sensory detection and escape capabilities among prey types are likely to contribute to the significant differences in capture success by the larval fish at any given age during the planktonic phase.

Each component of the sensori-motor system of the prey will influence the outcome of a predator-prey interaction. However, the relative importance of sensory detection, response latency and escape behavior is likely to change with development of the prey and the predator. Furthermore, copepod escape responses differ among species^36^,^82^, and some of these differences are already present in nauplii^41^,^83^. While *P. crassirostris* co-occurs with clownfish larvae, it is nevertheless, only one of the common species found in coastal pelagic environments^58^,^84^. Little is known about how species-specific differences in copepod escape response might affect the diet of coral reef fishes, however, there is evidence of selective feeding in larvae of Atlantic mackerel even at the copepod nauplius level^71^. Specifically, gut content analysis suggested that mackerel selected for the nauplii of two species, but against those of the third species.

The Manly-Chesson Selectivity Index is used extensively as an indicator of whether a particular prey is consumed in proportion to its occurrence^85^. However, in general, this index is based on gut content analysis, and as a result does not distinguish between encounter probability, selectivity, and capture success^69^. Here, we applied the Manly-Chesson Index to specifically address the predator’s selectivity for prey type, independent of capture success. Thus, it addresses the question of whether the clownfish larvae preferentially strike or avoid certain prey types as a function of age during the planktonic phase. Fish larvae that were too young to capture the adult copepods showed low selectivity for this prey type, while showing preference for the nauplii. Selectivity for nauplii decreased steadily with larval age. Based on the observation that no prey were rejected in our experimental conditions, relative feeding rates of *A. ocellaris* on the three prey types can be calculated by multiplying capture success by the selectivity index. Thus, numerically, nauplii constituted 100% of the diet of *A. ocellaris* larvae at first feeding, and this percentage dropped to 50% at 14 dph, while the relative number of copepodites and adults increased with age reaching an estimated 33% and 17%, respectively.

The trends we observed in strike selectivity and capture success would suggest that the contribution of nauplii to the diet would continue to decrease with increasing size of fish larvae. Gut content analyses of larger Pomacentridae (≥8 mm) are consistent with this prediction. While nauplii were still consumed, copepodites constituted the majority of the gut contents (84%)^9^, with positive selectivity for small copepods (copepodites) and avoidance of nauplii^18^. Given the difference in nutrition (~30-fold difference in dry weight between nauplii and adult *P. crassirostris*), fish larvae would be better off preying on adults than nauplii even with a lower capture success rate. However, encounter rates with nauplii may be much greater in the natural environment, given that the younger life stages typically outnumber copepodites and adults^86^. Thus, given the higher capture success and higher abundances of nauplii, low numbers of nauplii in fish guts would suggest avoidance of these small prey as larval fish become larger.

In this study, we focused on predatory success during the early larval fish phase of the false percula clownfish, *A. ocellaris*. Predator-prey experiments performed with larval fish between 1 and 14 dph and different developmental stages of copepod prey (*P. crassirostris* nauplii, copepodites and adult copepods) determined that initial capture success was low and limited to nauplii, the least evasive of the three prey types. Predatory abilities showed incremental improvement in capture success occurring at daily intervals with the addition of more evasive prey at 3 and 8 dph and increases in capture success for all prey types. While copepods are the most common prey of larval fishes, species differ in their swimming and escape behaviors^36^,^41^. We tested a single species of copepod, yet in their habitat fish larvae will encounter a diverse array of copepod species as well as developmental stages. While adult fishes modulate their predatory strategy in response to prey type^82^,^87^, the ability for larval fishes to do so may be more limited. Thus, differences in capture success for different copepod species could contribute to prey selectivity that is calculated from gut contents.

## Materials and Methods

### Cultivation P. crassirostris

*P. crassirostris* were cultured following dilution and algal feeding protocols developed for a related paracalanid, *Bestiolina similis*^88^,^89^,^90^. Briefly, multiple high-density (up to 10 individuals ml^−1^) copepod cultures were maintained in 20 L containers. *Tisochrysis lutea* (formerly known as *Isochrysis galbana* Tahitian strain) was added daily and phytoplankton densities in the copepod cultures were maintained between 10^4^ and 10^5^ cells ml^−1^. In order to match the feeding needs of *A. ocellaris* larvae, *P. crassirostris* cultures were managed for the production of nauplii and copepodites by timing peaks in *P. crassirostris* reproduction, which occurred shortly after dilution of the stock culture^89^. Phytoplankton and copepods were maintained in seawater (35 ppt), constantly aerated and kept under a 12:12 hour light:dark cycle at ambient temperature (21–26 °C). Lighting was provided with 20-watt fluorescent lights (T12/Daylight).

### Preparation of experimental prey fields

*P. crassirostris* were sorted by sieving using pairs of sieves with coarser and finer mesh sizes to isolate the target prey. The first pair, with 35 and 100 μm Nitex mesh sizes, concentrated nauplii of stages NI to NIV, which ranged in length between 60–130 μm in length and 40–60 μm in width^40^,^89^,^90^. The second pair, with mesh sizes of 200 and 275 μm, isolated copepodites (CI to CIII), which ranged in size between 200–300 μm in length and 80–110 μm in width^40^,^90^. The third pair, with mesh sizes of 323 and 400 μm, concentrated adult females (length: 400 μm, width: 160 μm)^40^,^90^. Prey samples were checked for the target stages and enumerated under a dissecting stereomicroscope (Olympus Corporation, Shinjuku, Tokyo, Japan; SZX-ILLB2) at 50× magnification. The concentrated *P. crassirostris* samples were then used to seed the observation chamber with either a single prey type at 1 individual ml^−1^ (experiment I), or 0.33 individuals ml^−1^ per prey type for the mixed prey trials (experiment II) (see below).

### Larval fish rearing protocol

Experimental data were obtained from *Amphiprion ocellaris* larvae reared in the laboratory. Two experimental series were completed using seven broods of ca. 200 eggs each from a breeding pair of *A. ocellaris* (Table 3). Eggs were obtained from a fish breeder on the day of hatching (0 days post hatch [dph]), allowed to hatch overnight, and transferred to a rearing container (20 L) maintained in a water bath kept at 25 to 27 °C. Fish larvae were fed daily on all life stages of *P. crassirostris* to target prey densities of 5 copepods ml^−1^. The daily feeding ration was usually depleted by the next morning, and daily consumption of prey by the fish larvae was estimated at 300 to 1000 copepods per individual per day depending on larval age and prey size. In addition, the rearing tank was seeded with *T. lutea* and phytoplankton densities were kept at 1 × 10^3^ cells ml^−1^. Fish handling and behavioral protocols were carried out in accordance with approved guidelines set by the Office of Research Compliance at the University of Hawaii (Hawaii, USA), and all experimental protocols were approved by the Institutional Animal Care & Use Committee (IACUC) for animal research under protocol number 1045. At 14 dph all surviving fish larvae (ca. 20–30%) were returned to the breeder.

### Morphological measurements of fish larvae

Larval length and gape width were measured from 1 to 14 dph at 1-day intervals. For each set, six *A. ocellaris* were euthanized from a single brood using a solution of 0.06 g/ml Ethyl 3-aminobenzoate methanesulfonate salt (MS222) (Sigma-Aldrich Inc., Saint Louis, MO, USA; catalog no. A5040-25G), preserved in a solution of 5% formalin in seawater, and measured for total length and jaw size within one week of fixation. Total length, a metric commonly used for clownfish larvae, is the greatest straight-line distance measured between the most anterior and posterior points of the body^91^,^92^. Measurements of fish larvae were made using a reticle calibrated with a 2.0 mm microscope micrometer at 12× for total length and 100× for mouth gape (Wild model M5A dissecting microscope). Upper jaw lengths were measured from the anterior-most point of the premaxilla to the posterior edge of the maxilla. Lower jaw lengths were measured from anterior-most part of the mandible to it posterior edge. Mouth gape was determined using Pythagorean theorem, assuming that the jaws represent two sides of a right triangle and the hypotenuse is the expected mouth gape^93^,^94^.

### Behavioral observations

The experimental design for the behavioral observations was close to the feeding conditions in the rearing tank in terms of prey numbers and fish density. Thus, the goal of these observations was to obtain quantitative data on larval fish capture success on prey upon which they had been reared. For each trial, 10 *A. ocellaris* larvae were transferred from the rearing tank into the observation chamber (18 × 18 × 10 cm aquarium made of Plexiglas) filled with 3 L of filtered seawater (Whatman filters, GF/C). The fish larvae were allowed to acclimate for 15 minutes, before adding the 3 × 10^3^ prey (1 ml^−1^). Each experimental trial lasted for 60 minutes and prey densities declined by 25% or less during this time. *A. ocellaris* and *P. crassirostris* interactions were filmed at 30 frames per second (fps) with a CCTV video camera (Panasonic Corporation, Kadoma, Osaka, Japan; model WV-BP310) equipped with a Nikkor 50 mm lens (Nikon Corporation, Shinjuku, Tokyo, Japan; model 1433) and recorded on a digital high definition videocassette recorder (Sony Corporation, Minato, Tokyo, Japan; model GV-HD700). The camera lens was positioned 0.3 m from the observation chamber and the lens was focused in a plane at the center with a field of view of 4 cm^2^. The container was uniformly illuminated from above with one 20-watt fluorescent light providing 1,900 lumens of light. Water temperature was between 23 and 25 °C.

Each day upon completion of filming, fish larvae were either fixed for length and gape measurements (see above), or they were returned to a second rearing container to avoid re-sampling. For the data analysis, videos were reviewed, larval predatory strikes identified and scored as to outcome (successful/unsuccessful). In a second analysis for experiment II, predatory strikes were scored for the targeted prey type to determine selectivity. For approximately 15% of strikes, resolution, clarity or contrast was insufficient to accurately determine prey type and these were excluded from the final dataset.

### Behavioral Experiments

Experimental details are summarized in Table 3. In Experiment I, larval fish were presented with one of the three prey types at three ages (1, 3 and 10 dph) to determine the ability of the predator to capture a particular prey type in the absence of other choices. The choice of ages included first feeding (1 dph), absorption of the yolk sac (3 dph) and post-flexion (10 dph). In these experimental conditions, the number of strikes scored for any individual trial increased with larval age, and the number of strikes per trial was greater than 10, so that more than one strike would have been recorded for an individual fish. For each combination of prey type and larval fish age, video footage was reviewed until 100 strikes with unambiguous outcomes were identified, and each strike was scored for capture success. Ambiguous outcomes were noted, but not included in subsequent data analysis. A total of 29 trials (290 fish larvae) were run to obtain 100 observations for each prey type. At 1 and 3 dph, the fish larvae attempted few strikes on adult copepods resulting in fewer than 100 observations for this prey type.

In experiment II, capture success was examined in the presence of mixed prey assemblages with equal densities of each prey type (Table 3) to determine changes in predatory ability at one-day intervals from 1 to 14 dph. In this experiment, 72 feeding trials (720 fish larvae) were completed using five separate broods (Table 3). As in experiment I, the number of strikes per trial was usually greater than 10, so that more than one strike would have been recorded for any individual fish. Because the frequency of strikes differed between trials, and in particular with age, the predatory ability of *A. ocellaris* was determined by scoring capture success of 100 strikes for any given prey type. Videotapes from experimental trials for a given larval age were reviewed and analyzed until the cumulative number of scored strikes reached 100. Fewer than 100 strikes were scored for larval fish attacking copepodites on 1 dph and adult prey on 1–6 dph because of low strike incidence.

In addition, the predator’s choice of prey (selectivity) was determined by analyzing strike frequency on each prey type. For the prey selectivity analysis, 100 strikes were chosen from a subset of strikes by using a random number generator to select a starting point (to the minute) amongst all of the footage recorded for each larval age and then reviewing the strikes in continuous order to determine the targeted prey type for each strike. Prey selectivity was computed as the Manly-Chesson selectivity index^85^:


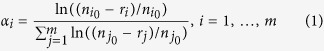


Where in Equation (1) r_*i*_ = the number of prey individuals of type *i* attacked, n_*i0*_ = the number of prey individuals of type *i* present at the beginning of each 60 min trial, m = the total number of prey types present and *j* signifies prey category since each prey category is added in series. This index accounts for the dynamic probabilities of prey encounter over the course of experimental trials without prey replacement. Use of the index requires the assumption that encounters with prey items, which do not result in consumption do not affect the predator’s subsequent behavior. Note that the application of the index here is unusual since it computes an index for prey strikes, not consumed prey^85^. Distinguishing between prey types was done by size.

### Statistical analysis

A logistic regression analysis was performed on the data with strike outcome as the dichotomous dependent variable and larval *A. ocellaris* age (Expt I: 1, 3 and 10 dph, Expt. II: 1–14 dph) and prey type (nauplii, copepodites and adults) as independent variables. The likelihood-ratio method was used to estimate probability values, and standard deviations for capture success for each prey and larval age combination were calculated based on a binomial distribution. A Pearson chi-square test was performed on the selectivity data to test the null hypothesis that strikes on each prey type occurred in proportion to the availability of each prey type in the environment, which was assumed to be 1/3 since prey types were presented in equal proportion. Statistical analyses were performed with the use of IBM SPSS version 19.0.

## Additional Information

**How to cite this article**: Jackson, J. M. and Lenz, P. H. Predator-prey interactions in the plankton: larval fish feeding on evasive copepods. *Sci. Rep.* **6**, 33585; doi: 10.1038/srep33585 (2016).

## Figures and Tables

**Table 1:** Statistical analysis for Experiments I and II.

Expt	Effect on attack outcome	df	p-value
Expt. I	Larval fish age	2	<0.005
	Prey type	2	<0.005
Expt. II	Larval fish age	13	<0.005
	Prey type	2	<0.005

The effects of age of fish larvae and prey type on attack outcome were tested using a logistic regression.

**Table 2:** The effect of prey types presented in experiment II on attack outcome for days 1 to 14, and days 7 to 14 post-hatch.

Age Interval	Attack outcome by prey type	df	p-value
Days 1–14	Nauplii	1	<0.005
	Copepodites	1	<0.005
	Adults	1	0.997*
Days 7–14	Nauplii	1	<0.005
	Copepodites	1	<0.005
	Adults	1	<0.005

^*^Days 1–14 post-hatch included no successful captures of adult prey during the initial 7 days.

**Table 3:** Summary of experimental conditions during Experiments I and II.

	Experiment I	Experiment II
Age (days post-hatch)	1, 3 & 10	1–14
Quantitative analysis	Capture success	Capture success Prey selectivity
Prey densities (ind ml^−1^)	1	1
Prey assemblages	Single prey/trial(NI-NIV) + (CI-CIII) + (CVI)	Mixed prey/trial(NI-NIV) + (CI-CIII) + CVI
Fish larvae (#/trial)	10	10
Broods (#/experiment)	2	5
60 min trials (#)	29	72
Time of day	10:00–13:00	10:00–16:00

## References

[b1] Houde E. D. Emerging from Hjort’s shadow. J Northw Atl Fish Sci 41, 53–70 (2008).

[b2] Bergenius M. A., Meekan M. G., Robertson R. D. & McCormick M. I. Larval growth predicts the recruitment success of a coral reef fish. Oecologia 131, 521–525 (2002). 10.1007/s00442-002-0918-4 28547546

[b3] Takasuka A., Oozeki Y., Kimura R., Kubota H. & Aoki I. Growth-selective predation hypothesis revisited for larval anchovy in offshore waters: cannibalism by juveniles versus predation by skipjack tunas. Mar Ecol Prog Ser 278, 297–302. (2004).

[b4] Rønnestad I. . Feeding behaviour and digestive physiology in larval fish: current knowledge, and gaps and bottlenecks in research. Rev Aquacult 5, S59–S98 (2013).

[b5] China V. & Holzman R. Hydrodynamic starvation in first-feeding larval fishes. Proc Natl Acad Sci USA 111, 8083–8088, doi: 10.1073/pnas.1323205111 (2014). 24843180 PMC4050599

[b6] Shulzitski K. . Close encounters with eddies: oceanographic features increase growth of larval reef fishes during their journey to the reef. Biol Lett 11 (2015). 10.1098/rsbl.2014.0746 PMC4321146 25631227

[b7] De Figueiredo G. M., Nash R. D. & Montagnes D. J. The role of the generally unrecognised microprey source as food for larval fish in the Irish Sea. Mar Biol 148, 395–404 (2005).

[b8] De Figueiredo G. M., Nash R. D. & Montagnes D. J. Do protozoa contribute significantly to the diet of larval fish in the Irish Sea? J Mar Biol Assoc UK 87, 843–850 (2007).

[b9] Sampey A., McKinnon A. D., Meekan M. G. & McCormick M. I. Glimpse into guts: overview of the feeding of larvae of tropical shorefishes. Mar Ecol Prog Ser 339, 243–257 (2007).

[b10] Llopiz J. K. & Cowen R. K. Variability in the trophic role of coral reef fish larvae in the oceanic plankton. Mar Ecol Prog Ser 381, 259–272 (2009).

[b11] Morote E., Olivar M. P., Villate F. & Uriarte I. A comparison of anchovy (*Engraulis encrasicolus*) and sardine (*Sardina pilchardus*) larvae feeding in the Northwest Mediterranean: influence of prey availability and ontogeny. ICES J Mar Sci 67, 897–908 (2010).

[b12] Llopiz J. K. Latitudinal and taxonomic patterns in the feeding ecologies of fish larvae: a literature synthesis. J Marine Syst 109 (2013).

[b13] Houde E. D. Comparative growth, mortality, and energetics of marine fish larvae: Temperature and implied latitudinal effects. Fish Bull 87, 471–495 (1989).

[b14] Houde S. E. L. & Roman M. R. Effects of food quality on the functional ingestion response of the copepod *Acartia tonsa*. Mar Ecol Prog Ser 40, 69–77 (1987).

[b15] Sponaugle S., Llopiz J. K., Havel L. N. & Rankin T. L. Spatial variation in larval growth and gut fullness in a coral reef fish. Mar Ecol Prog Ser 383, 239–249 (2009).

[b16] Leis J. M. & Clark D. L. Feeding greatly enhances swimming endurance of settlement-stage reef-fish larvae of damselfishes (Pomacentridae). Ichthyol Res 52, 185–188 (2005).

[b17] Lough R. G. & Mountain D. G. Effect of small-scale turbulence on feeding rates of larval cod and haddock in stratified water on Georges Bank. Deep-Sea Res Pt II 43, 1745–1772 (1996).

[b18] Carassou L., Le Borgne R. & Ponton D. Diet of pre-settlement larvae of coral-reef fishes: selection of prey types and sizes. J Fish Biol 75, 707–715 (2009). 20738568 10.1111/j.1095-8649.2009.02312.x

[b19] Østergaard P., Munk P. & Janekarn V. Contrasting feeding patterns among species of fish larvae from the tropical Andaman Sea. Mar Biol 146, 595–606 (2005).

[b20] Munk P. & Kiørboe T. Feeding behaviour and swimming activity of larval herring (*Clupea harengus*) in relation to density of copepod nauplii. Mar Ecol Prog Ser 24, 15–21 (1985).

[b21] Von Herbing I. H. & Gallager S. M. Foraging behavior in early Atlantic cod larvae (*Gadus morhua*) feeding on a protozoan (*Balanion* sp.) and a copepod nauplius (*Pseudodiaptomus* sp.). Mar Biol 136, 591–602 (2000).

[b22] Rosenthal H. Untersuchungen über das Beutefangverhalten bei Larven des Herings *Clupea harengus*. Mar Biol 3, 208–221 (1969).

[b23] Rosenthal H. & Hempel G. In Marine Food Chains (ed Steele J. H.) 344–364 (University of California Press, 1970).

[b24] Runge J. A. Should we expect a relationship between primary production and fisheries? The role of copepod dynamics as a filter of trophic variability. Hydrobiologia 167, 61–71 (1988).

[b25] Huys R. & Boxshall G. A. Copepod evolution (The Ray Society, Unwin Brothers, 1991).

[b26] Humes A. G. How many copepods? Hydrobiologia, 292, 1–7 (1994).

[b27] Buskey E. J., Lenz P. H. & Hartline D. K. Sensory perception, neurobiology and behavioral adaptations for predator avoidance in planktonic copepods. Adapt Behav 20, 57–66 (2012).

[b28] Strickler J. R. & Bal A. K. Setae of the first antennae of the copepod *Cyclops scutifer* (Sars): Their structure and importance. Proc Natl Acad Sci USA 70, 2656–2659 (1973). 16592109 10.1073/pnas.70.9.2656 PMC427076

[b29] Yen J., Lenz P. H., Gassie D. V. & Hartline D. K. Mechanoreception in marine copepods - electrophysiological studies on the 1^st^ antennae. J Plankton Res 14, 495–512 (1992).

[b30] Hartline D. K., Lenz P. H. & Herren C. M. Physiological and behavioral studies of escape responses in calanoid copepods. Mar Freshw Behav Phy 27, 199–212 (1996).

[b31] Lenz P. H. & Hartline D. K. In Nervous Systems and Control of Behavior The Natural History of the Crustacea (eds Derby C. & Thiel M.) Ch. 11, 293–320 (Oxford University Press, 2014).

[b32] Weatherby T. M. & Lenz P. H. Mechanoreceptors in calanoid copepods: designed for high sensitivity. Arthropod Struct Dev 29, 275–288 (2000). 18088933 10.1016/s1467-8039(01)00011-1

[b33] Fields D. M., Shaeffer D. S. & Weissburg M. J. Mechanical and neural responses from the mechanosensory hairs on the antennule of *Gaussia princeps*. Mar Ecol Prog Ser 227, 173–186 (2002).

[b34] Strickler J. R. In Swimming and Flying in Nature (eds Wu T. Y.-T., Brokaw C. J. & Brennan C.) 599–613 (Plenum Press, 1975).

[b35] Buskey E. J., Lenz P. H. & Hartline D. K. Escape behavior of planktonic copepods in response to hydrodynamic disturbances: high speed video analysis. Mar Ecol Prog Ser 235, 135–146 (2002).

[b36] Burdick D. S., Hartline D. K. & Lenz P. H. Escape strategies in co-occurring calanoid copepods. Limnol Oceanogr 52, 2373–2385 (2007).

[b37] Lenz P. H. & Hartline D. K. Reaction times and force production during escape behavior of a calanoid copepod, Undinula vulgaris. Mar Biol 133, 249–258, doi: 10.1007/S002270050464 (1999).

[b38] Lenz P. H., Hower A. E. & Hartline D. K. Force production during pereiopod power strokes in *Calanus finmarchicus*. J Marine Syst 49, 133–144, doi: 10.1016/J.Jmarsys.2003.05.006 (2004).

[b39] Lenz P. H., Hartline D. K. & Davis A. D. The need for speed. I. Fast reactions and myelinated axons in copepods. J Comp Physiol A 186, 337–345 (2000). 10798722 10.1007/s003590050434

[b40] McKinnon A. D. . The potential of tropical paracalanid copepods as live feeds in aquaculture. Aquaculture 223, 89–106 (2003).

[b41] Bradley C. J., Strickler J. R., Buskey E. J. & Lenz P. H. Swimming and escape behavior in two species of calanoid copepods from nauplius to adult. J Plankton Res 35, 49–65, doi: 10.1093/Plankt/Fbs088 (2013).

[b42] Williams P. J., Brown J. A., Gotceitas V. & Pepin P. Developmental changes in escape response performance of five species of marine larval fish. Can J Fish Aquat Sci 53, 1246–1253 (1996).

[b43] Shepherd T. D., Costain K. E. & Litvak M. K. Effect of development rate on the swimming, escape responses and morphology of yolk-sac stage larval American plaice *Hippoglossoides platessoides*. Mar Biol 137, 737–745 (2000).

[b44] Hunter J. R. Swimming and feeding behavior of larval anchovy *Engraulis mordax*. Fish Bull 70, 821–838 (1972).

[b45] Gerritsen J. & Strickler J. R. Encounter probabilities and community structure in zooplankton: a mathematical model. J Fish Res Board Can 34, 73–82 (1977).

[b46] Lasker R. Marine fish larvae: morphology, ecology, and relation to fisheries 87 (Sea Grant Program, Seattle: Washington, 1981).

[b47] Coughlin D. J. Suction prey capture by clownfish larvae (*Amphiprion perideraion*). Copeia 1994, 242–246 (1994).

[b48] Wellington G. M. & Victor B. C. Planktonic larval duration of one hundred species of Pacific and Atlantic damselfishes (Pomacentridae). Mar Biol 101, 557–567 (1989).

[b49] Green B. S. & McCormick M. I. Ontogeny of the digestive and feeding systems in the anemonefish *Amphiprion melanopus*. Environ Biol Fishes 61, 73–83 (2001).

[b50] Liew H. J., Ambak M. A. & Abol-Munafi A. B. False Clownfish - Breeding, Behavioral, Embryonic and Larval Development, Rearing and Management of Clownfish Under Captivity (LAP Lambert Academic Publishing, 2010).

[b51] Coughlin D. J., Strickler J. R. & Sanderson B. Swimming and search behaviour in clownfish, *Amphiprion perideraion*, larvae. Anim Behav 44, 427–440 (1992).

[b52] Kumar, T., A. & Balasubramanian T. Broodstock development, spawning and larval rearing of the false clownfish, *Amphiprion ocellaris* in captivity using estuarine water. Curr Sci India 97, 1483–1486 (2002).

[b53] Turner J. T. The annual cycle of zooplankton in a Long Island Estuary. Estuaries 5, 261–274 (1982).

[b54] Hopcroft R. R., Roff J. C. & Lombard D. Production of tropical copepods in Kingston Harbour, Jamaica: the importance of small species. Mar Biol 130, 593–604 (1998).

[b55] Lo W. T., Chung C. L. & Shih C. T. Seasonal distribution of copepods in Tapong Bay, southwestern Taiwan. Zool Stud 43, 464–474 (2004).

[b56] Hoover R. S. . Zooplankton response to storm runoff in a tropical estuary: bottom-up and top-down controls. Mar Ecol Prog Ser 318, 187–201 (2006).

[b57] Duggan S., McKinnon A. D. & Carleton J. H. Zooplankton in an Australian tropical estuary. Estuar Coast 31, 455–467 (2008).

[b58] McKinnon A. D., Duggan S., Carleton J. H. & Böttger-Schnack R. Summer planktonic copepod communities of Australia’s North West Cape (Indian Ocean) during the 1997–1999 El Niño/La Niña. J Plankton Res 30, 839–855 (2008).

[b59] McKinnon A. D., Duggan S. & De’ath G. Mesozooplankton dynamics in nearshore waters of the Great Barrier Reef. Estuar Coast Shelf S 63, 497–511 (2005).

[b60] Jungbluth M. J. & Lenz P. H. Copepod diversity in a subtropical bay based on a fragment of the mitochondrial COI gene. J Plankton Res 35, 630–643, doi: 10.1093/Plankt/Fbt015 (2013).

[b61] Lenz P. H. The biogeography and ecology of myelin in marine copepods. J Plankton Res 34, 575–589, doi: 10.1093/Plankt/Fbs037 (2012).

[b62] Timm J. & Kochzius M. Geological history and oceanography of the Indo‐Malay Archipelago shape the genetic population structure in the false clown anemonefish (*Amphiprion ocellaris*). Mol Ecol 17, 3999–4014 (2008). 19238702 10.1111/j.1365-294x.2008.03881.x

[b63] Cornils A. & Blanco-Bercial L. Phylogeny of the Paracalanidae giesbrecht, 1888 (Crustacea: Copepoda: Calanoida). Mol Phylogenet Evol 69, 661–672 (2013). 10.1016/j.ympev.2013.06.018 23831457

[b64] Schmitt R. J. & Holbrook S. J. Gape-limitation, foraging tactics and prey size selectivity of two microcarnivorous species of fish. Oecologia, 63, 6–12 (1984). 10.1007/BF00379778 28311159

[b65] Bremigan M. T. & Stein R. A. Gape-dependent larval foraging and zooplankton size: implications for fish recruitment across systems. Can J Fish Aquat Sci 51, 913–922 (1994).

[b66] DeVries D. R., Stein R. A. & Bremigan M. T. Prey selection by larval fishes as influenced by available zooplankton and gape limitation. T Am Fish Soc 127, 1040–1050 (1998).

[b67] Gill A. B. The dynamics of prey choice in fish: the importance of prey size and satiation. J Fish Biol 63, 105–116 (2003).

[b68] Holling C. S. The functional response of predators to prey density and its role in mimicry and population regulation. Mem Entomol Soc Can 97, 5–60 (1965).

[b69] Drenner R. W., Strickler J. R. & O’Brien W. J. Capture probability: The role of zooplankter escape in the selective feeding of planktivorous fish. J Fish Res Board Can 35, 1370–1373 (1978).

[b70] Houde E. D. & Schekter R. C. Feeding by marine fish larvae: developmental and functional responses. Environ Biol Fishes 5, 315–334 (1980).

[b71] Peterson W. T. & Ausubel S. J. Diets and selective feeding by larvae of Atlantic mackerel *Scomber scombrus* on zooplankton. Mar Ecol Prog Ser 17, 65–75 (1984).

[b72] Russo T., Costa C. & Cataudella S. Correspondence between shape and feeding habit changes throughout ontogeny of gilthead sea bream *Sparus aurata* L., 1758. J Fish Biol 71, 629–656 (2007).

[b73] Jones W. R. & Janssen J. Lateral line development and feeding behavior in the mottled sculpin, *Cottus bairdi* (Scorpaeniformes: Cottidae). Copeia 1992, 485–492 (1992).

[b74] Yúfera M. & Darias M. J. The onset of exogenous feeding in marine fish larvae. Aquaculture 268, 53–63 (2007).

[b75] Scharf F. S., Juanes F. & Rountree R. A. Predator size-prey size relationships of marine fish predators: interspecific variation and effects of ontogeny and body size on trophic-niche breadth. Mar Ecol Prog Ser 208, 229–248 (2000).

[b76] Krebs J. M. & Turingan R. G. Intraspecific variation in gape–prey size relationships and feeding success during early ontogeny in red drum. Sciaenops ocellatus. Environ Biol Fishes 66, 75–84 (2003).

[b77] Lenz P. H., Weatherby T. M., Weber W. & Wong K. K. Sensory specialization along the first antenna of a calanoid copepod, *Pleuromamma xiphias* (Crustacea). Mar Freshw Behav Phy 27, 213–221 (1996).

[b78] Davis A. D., Weatherby T. M., Hartline D. K. & Lenz P. H. Myelin-like sheaths in copepod axons. Nature (London) 398, 571–571 (1999). 10217140 10.1038/19212

[b79] Domenici P. The scaling of locomotor performance in predator–prey encounters: from fish to killer whales. Comp Biochem Physiol A Mol Integr Physiol 131, 169–182 (2001). 11733175 10.1016/s1095-6433(01)00465-2

[b80] Clarke R. D., Buskey E. J. & Marsden K. C. Effects of water motion and prey behavior on zooplankton capture by two coral reef fishes. Mar Biol 146, 1145–1155 (2005).

[b81] Gemmell B. J., Sheng J. & Buskey E. J. Morphology of seahorse head hydrodynamically aids in capture of evasive prey. Nat Commun 4 (2013). 10.1038/ncomms3840 24281430

[b82] Waggett R. J. & Buskey E. J. Calanoid copepod escape behavior in response to a visual predator. Mar Biol 150, 599–607 (2007).

[b83] Titelman J. & Kiørboe T. Predator avoidance by nauplii. Mar Ecol Prog Ser 247, 137–149 (2003).

[b84] McKinnon A. D. & Duggan S. Summer copepod production in subtropical waters adjacent to Australia’s North West Cape. Mar Biol 143, 897–907 (2003).

[b85] Chesson J. The estimation and analysis of preference and its relatioship to foraging models. Ecology 64, 1297–1304 (1983).

[b86] Turner J. T. The importance of small planktonic copepods and their roles in pelagic marine food webs. Zool Stud 43, 255–266 (2004).

[b87] Nemeth D. Modulation of attack behavior and its effect on feeding performance in a trophic generalist fish. J Exp Biol 200, 2155–2164 (1997). 9320068 10.1242/jeb.200.15.2155

[b88] VanderLugt K., Cooney M. J., Lechner A. & Lenz P. H. Cultivation of the paracalanid copepod, *Bestiolina similis* (Calanoida: Crustacea). J World Aquacult Soc 40, 616–628 (2009).

[b89] VanderLugt K. & Lenz P. H. Management of nauplius production in the paracalanid, *Bestiolina similis* (Crustacea: Copepoda): effects of stocking densities and culture dilution. Aquaculture 276, 69–77 (2008).

[b90] Jackson J. M. Larval clownfish Amphiprion ocellaris predatory success and selectivity when preying on the calanoid copepod Parvocalanus crassirostris M.S. thesis, University of Hawaii at Manoa, (2011).

[b91] Frakes T. & Hoff F. H. Effect of high nitrate-N on the growth and survival of juvenile and larval anemonefish, Amphiprion ocellaris. Aquaculture 29, 155–158 (1982).

[b92] Arvedlund M., McCormick M. I. & Ainsworth T. Effects of photoperiod on growth of larvae and juveniles of the anemonefish *Amphiprion melanopus*. Naga, The ICLARM Quarterly 23, 18–23 (2000).

[b93] Shirota A. Studies on the mouth size of fish larvae. Bull Jpn Soc Sci Fish 36, 353–367 (1970).

[b94] Guma’a S. A. The food and feeding habits of young perch, *Perca fluviatilis*, in Windermere. Freshw Biol 8, 177–187 (1978).

